# Quality of life themes in Canadian adults and street youth who are homeless or hard-to-house: A multi-site focus group study

**DOI:** 10.1186/1477-7525-10-93

**Published:** 2012-08-15

**Authors:** Anita Palepu, Anita M Hubley, Lara B Russell, Anne M Gadermann, Mary Chinni

**Affiliations:** 1 Centre for Health Evaluation and Outcome Sciences, St. Paul’s Hospital & University of British Columbia, 620B-1081 Burrard Street, Vancouver, BC, V6Z 1Y6, Canada; 2 Department of ECPS, University of British Columbia, 2125 Main Mall, Vancouver, BC, V6T 1Z4, Canada

**Keywords:** Homeless, Quality of life, Hard-to-house

## Abstract

**Background:**

The aim of this study was to identify what is most important to the quality of life (QoL) of those who experience homelessness by directly soliciting the views of homeless and hard-to-house Canadians themselves. These individuals live within a unique social context that differs considerably from that of the general population. To understand the life areas that are most important to them, it is critical to have direct input from target populations of homeless and hard-to-house persons.

**Methods:**

Focus groups were conducted with 140 individuals aged 15 to 73 years who were homeless or hard-to-house to explore the circumstances in which they were living and to capture what they find to be important and relevant domains of QoL. Participants were recruited in Toronto, Ottawa, Montreal, and Vancouver. Content analysis was used to analyze the data.

**Results:**

Six major content themes emerged: Health/health care; Living conditions; Financial situation; Employment situation; Relationships; and Recreational and leisure activities. These themes were linked to broader concepts that included having choices, stability, respect, and the same rights as other members of society.

**Conclusions:**

These findings not only aid our understanding of QoL in this group, but may be used to develop measures that capture QoL in this population and help programs and policies become more effective in improving the life situation for persons who are homeless and hard-to-house.

Quality of life themes in Canadian adults and street youth who are homeless or hard-to-house: A multi-site focus group study.

## Introduction

The World Health Organization (WHO) defines quality of life (QoL) as “an individual’s perceptions of their position in life in the context of the culture and value systems in which they live, and in relation to their goals, expectations, and concerns” (p. 13) [1]. This definition of QoL highlights the factors that impact subjective evaluations of life circumstances and may explain the apparent discrepancies sometimes found between an objective evaluation of a person’s life circumstances and his/her own self-evaluation [2].

Despite statistics estimating the number of homeless to be in the millions in the United States and Europe [3,4], a review of the literature on subjective QoL in individuals who are homeless [5] revealed that surprisingly little research has focused on the subjective QoL of individuals who are homeless or hard-to-house. This review found that homeless individuals tended to have lower QoL than people who are housed. Lower QoL was also found to be associated with poorer mental health, substance misuse, and being male. Unfortunately, much of our understanding of the relationships between QoL and other variables (including demographic and health variables) is based on very limited information.

Moreover, no studies appear to have asked individuals who are homeless or hard-to-house about what is important to, or impacts, their QoL [5]. This leaves a tremendous gap in our understanding of QoL in this population and the role such information may have on our ability to effectively improve the QoL of individuals who are homeless or hard-to-house. The population of individuals who are homeless and hard-to-house is diverse with individuals from various backgrounds and life situations. The lives of many homeless and hard-to-house individuals often contain many challenges such as past traumatic events (e.g., child abuse, rape, exposure to violence) [6], addictions [7], losses (e.g., financial, employment) [8], mental illness (e.g., depression, anxiety, schizophrenia) [9], chronic health conditions (e.g., hypertension, diabetes, head injuries, Hepatitis C, HIV/AIDS) [10-12], lack of affordable, safe and stable housing [13], and prejudice [9,14].

In addition, some individuals may have experienced adverse life circumstances and housing instability from an early age whereas for others critical life events and/or mental health problems may have triggered housing instability later in life, as illustrated by the following vignettes of homeless or hard-to-house individuals living in Vancouver.

Miriam, a 35-year old woman from a rural community, ran away from an abusive home at the age of nine. She has been working in the sex trade industry ever since. She lives with her husband who is physically abusive and demands that she works the street to support both of their addictions. Miriam has 5 children who were all taken away by Child and Family Services.

Bill is a 27-year old man who has just recently been housed in an apartment subsidized by the province. He has been diagnosed with bipolar disorder and dissociative disorder. Bill has been living on and off the streets for the last 6 years. He recently connected with mental health services and now feels more stable than he has been in a long time and is now on psychotropic medication.

Theresa is a 45-year old woman who came from a wealthy family and had a successful business with her husband. She suffered from alcohol dependence during her early 20s, but when she had children she recovered. After her children left home and her husband passed away she started drinking again and using cocaine until all the money was spent. She currently lives at a women's shelter in a low-income neighborhood and still battles her addiction. She tells friends that she moved to another city and her children that she 'just wants to live simply'. No one close to her knows the truth about her housing situation.

If QoL reflects an individual’s subjective perceptions of his/her position in life in the context of the culture and value systems in which he or she lives, it is important to understand the perspective of this group by gathering information directly from them. The importance of understanding QoL from, and including the voices of, one’s target population has been recognized in studies of QoL in other groups [15-18]. In these studies, more in-depth discussion and the use of focus groups is emphasized (over survey methodology, for example) as a way of encouraging discussion and obtaining more in-depth information about QoL.

In order to adequately understand QoL in homeless and hard-to-house individuals, it seems reasonable to build on the limited QoL literature related to this group by discussing QoL directly with them. Thus, we conducted focus groups with adults and youth who are homeless or hard-to-house in four Canadian cities to explore QoL and ask about the life areas most important to them.

## Methods

### Ethical approval

The University of British Columbia Behavioural Research Ethics Board, the St. Michael’s Hospital Research Ethics Board, the University of Ottawa Research Ethics Board and l*e comité d'éthique de la recherche en santé chez l'humain du Centre Hospitalier Universitaire de Sherbrooke et de l'Université de Sherbrooke* approved this study and the data collection in Vancouver, Toronto, Ottawa and Montreal, respectively.

### Settings and participants

Study participants were 140 homeless or hard-to-house individuals who were recruited from projects or sites in four large Canadian cities: Toronto, Ottawa, Montreal, and Vancouver. Being homeless was defined as sleeping in a homeless shelter, outside, in a park, abandoned building, train or bus station, vehicle, or other place not intended for human habitation for at least one night in the last 7 days or having had to sleep at a friend’s or relative’s place because the person did not have a place of his/her own. Persons who were “hard to house” were those who had a history of homelessness and were now residing in low income, supportive housing.

Participants were recruited from the following four projects or sites when they attended their next scheduled appointment:

### Toronto homeless health care utilization cohort

This large cohort study was designed to examine the factors associated with health care use among homeless persons in Toronto. The study involved 1200 (400 single males, 400 single females, and 400 mothers accompanied by dependent children) homeless participants between 2004–6 at meal programs or at homeless shelters for youth, adults, and families.

### Ottawa inner city health project

The Ottawa Inner City Health Project works collaboratively with service providers from health, housing, social services, and the legal system to address the health and housing needs of the chronically homeless. They have established a small number of residential services located in special facilities within the shelters for the homeless that include: a 15 bed Home Hospice where palliative care can be delivered; a 20 bed convalescent/infirmary facility; a 20 bed Management of Alcohol program for street alcoholics; and services to support 10 individuals in supportive housing. They admit, and deliver services to, approximately 150 patients per year, serving 50–60 at any given time.

### Montreal street youth cohort

This cohort study recruited 694 predominantly French-speaking subjects (68% male) between 2001 and 2003 to examine the residential trajectory of street-involved youth, risk-taking behaviors and health outcomes. The mean age at recruitment was 20.5 years. Participants were first homeless at a mean age of 15.5 years and they had been homeless for, on average, 1.6 years.

### Portland hotel society

The Portland Hotel Society (PHS) has served people in Vancouver’s Downtown Eastside and adjacent communities since 1993. Most residents of the PHS have been labeled “hard to house” and typically are dually-diagnosed with major mental illness and drug and alcohol dependencies. Home support services, home care nursing, visits from a primary care physician, medication delivery, methadone maintenance, meal programs, a needle exchange, and HIV outreach are available on-site at many of the single room occupancy hotels that they operate.

Excluded persons from the focus groups were those who were not homeless, hard to house, or in the case of Montreal not street-youth. Of the individuals invited to participate, those who were not willing to participate, or those who were not able to participate in the focus group due to speech difficulties or who did not speak English (in Vancouver, Ottawa, Toronto) or French (in Montreal) were excluded. Individuals with literacy challenges were included in the study and offered help in writing about the things that were important to their QOL.

### Moderators/Interviewers

Focus group moderators consisted of service providers or research assistants at each of the sites where participants were recruited. A standardized protocol was used with the moderators at each site. All were provided with a semi-structured interview guide and all had prior experience in (a) interviewing or moderating, and (b) working with either individuals who are homeless or vulnerable populations. Different moderators were used at the various sites because of: (a) the difficulty and cost of obtaining a moderator who could travel to all four cities (and could speak French for the Montreal site), and (b) the intention to use staff familiar to the study participants whenever possible to assist with building rapport and trust. Moderators used probes to elicit further information and obtain more detailed responses by the participants. In Vancouver, Ottawa and Montreal there were two moderators for each focus group and in Toronto, there was only one due to research staff availability at that time.

Focus groups were used to provide the opportunity for interaction among members of the group allowing them to question each other or explain opinions to one another [19]. This negotiation of meaning is especially relevant for a topic such as QoL as it allows exploring this construct in its diverse facets, which might not all have come up in individual interviews. Furthermore, compared to individual interviews, the use of focus groups can allow the inclusion of a larger number of participants to reflect a wide variety of personal experiences.

### Procedure

At each research site, staff asked potential participants during a regular visit if they were willing to return and participate in a group discussion. Focus groups were targeted to consist of 4 to 8 participants to ensure diversity of perceptions and, at the same time to provide the opportunity for everyone to share ideas and make the groups feasible in terms of logistics [20]. One focus group had only three participants because the additional participants did not attend that particular focus group.

The focus group sessions were conducted in places that were familiar to the participants. Each focus group session was audio taped and lasted 45 to 90 minutes. Eight focus groups were conducted in Toronto, six in Montreal, and five each in Ottawa and Vancouver. The overall aim of the focus groups was to provide an environment in which participants felt free to discuss important areas in their life and for the moderators to be open to the topics generated by the group. There was no attempt to guide or to lead participants in any given direction, area or topic. Great care and effort was undertaken to ensure that participants understood the aim and methods of the study. All participants received oral and written information about the study and signed an informed consent form. Participants received an honorarium of $20 Canadian dollars for their participation and lunch was provided.

In the focus groups, participants were first asked to write down anything they felt was important to their QoL. Participants were instructed not to censor their thoughts and to write down anything that came to mind. Once this task was completed, a discussion began during which participants were asked to read and discuss what they had recorded. Follow-up questions directed to each of the focus groups differed based on the ideas generated. Examples of questions were: what things in life bring you happiness?; what do you need to have a good life and feel safe?; what areas have a positive or a negative impact on your life?; what is important for your life to go well?; and what is important for your quality of life?. Participants were able to amend their lists throughout the discussion if they were reminded of areas that were important to them that they had not originally considered. At the end of the discussion, participants were asked to review their lists and choose the top five areas that were most important to their QoL, and rank them in order of importance (with ‘1’ being the most significant).

### Transcription and analysis of focus groups

Audiotapes of the focus group sessions were transcribed concurrently with the data collection. The third author, a graduate student with experience in working with homeless individuals and who also co-moderated the Vancouver focus groups, listened to all of the English-language recordings and noted significant themes relating to QoL in a written document, along with quotations supporting these themes. The transcription was guided by this list as the person transcribing noted whenever something was mentioned related to those topics during the focus group, but also when new topics emerged. In the case of the French-speaking focus groups at the Montreal site, a similar document was prepared by an individual who was fluently bilingual in English and French. Data from both the English and French groups were coded using QSR’s NVivo 2 qualitative software and subjected to a thematic analysis. Analysis focused on identifying the life areas that were most important to the participants’ QoL. The fourth author reviewed the coding of the transcripts. If there was disagreement with regard to the coding, it was discussed among the co-authors until consensus was achieved. Street youth and homeless adults were not distinguished in our analysis because the same themes were consistently identified. Quotes were chosen from the participants that best exemplified the life area theme that was discussed.

## Results

### Participants

Participants consisted of a total of 140 homeless and hard-to-house individuals living in four Canadian cities (Toronto, Ottawa, Montreal, Vancouver) – see Table 1. There were 97 men (69.3%) and 43 women (30.7%) aged 15 to 73 years, with a mean age of 32 years (SD = 14.8 years). The women in the sample (M = 27, SD = 10.5, range = 15–47) were, on average, younger than the men (M = 34; SD = 15.9, range = 15–73).

**Table 1:** Characteristics of Homeless and Hard-toHouse Individuals

**Variable**	**n (%**^**a**^**) or mean (SD)**
Site^b^	
Vancouver	27 (19.3)
Toronto	48 (34.3)
Ottawa	17 (12.1)
Montreal	48 (34.3)
Gender^b^	
Female	43 (30.7)
Male	97 (69.3)
Ethnic background^c^	
‘Canadian’	49 (53.9)
European descent	24 (26.4)
Aboriginal	12 (13.2)
Other	6 (6.5)
Age^b^	32 (14.8)
Education^d^	
Elementary only	22 (24.4)
Some high school	55 (61.2)
Some post-secondary	13 (14.4)
Current living situation^e^	
Housing designated for homeless	38 (43.2)
Homeless Shelter	35 (39.8)
Street (no shelter)	11 (12.5)
Market housing	4 (4.5)
Employment^c^	
Unemployed	67 (73.6)
Working casual or part-time	12 (13.2)
Volunteer or unpaid work	10 (11.0)
Retired	2 (2.2)

^a^Based only on respondents who provided information on the respective variable. There were missing data for several of the demographic questions. ^b^N = 140. ^c^n = 91. ^d^n = 90. ^e^n = 88 (not reported by street youth sample).

Of those who reported ethnic or cultural background, most participants would only self-identify as ‘Canadian’ (53.9%). Those self-identifying as being of European descent formed the second largest group at 26.4% and just over 13% of participants indicated that they were Aboriginal. Almost half (43.2%) of participants who provided information about their housing situation were currently living in housing designated for the homeless or hard-to-house, while another 39.8% were living in shelters, 12.5% were living on the street or without any shelter, and 4.5% were currently in market housing. Most participants (73.6%) were unemployed, while 13.2% were working casual or part-time, and 11.0% were engaged in volunteer or unpaid work. The majority of participants (61.2%) had some high school education, and 14.4% pursued some post-secondary education. Close to a quarter (24.4%) of those reporting education had ended their education after elementary school.

### Content themes

Six major content themes were identified in the content analysis: health/health care, living conditions, financial situation, employment situation, relationships, and recreational and leisure activities. Overall, these themes were common to both youths and adults.

### Health/Health care

Health and access to health care were clearly very important to many participants. Both youth and adult participants commented on the importance of being physically fit and mentally healthy. Individuals who were healthy were grateful to be so, whereas participants with health problems noted that these had a strong negative impact on their lives and that “*it’s hard to be happy if you’re not healthy*”. Being drug addicted and having limited or no access to drug treatment programs but direct access to all forms of street drugs was the topic of many discussions. Many participants discussed living with HIV. Participants also discussed the lack of relationship with healthcare providers, not enough proper assessment of needs, and lack of regular monitoring of medication. Participants reported that medical practitioners often did not seem to know their medical histories and offered little assistance in navigating the healthcare system. One participant expressed frustration that health services are only available a few days a week, and that therefore “*you have to have your emergencies on certain days*.”

The need to be close to a hospital or clinic was also important, as well as having a doctor or nurse to contact: “*Having a doctor or nurse around 24 hours a day. That’s important to me. I’m getting sicker*” and “*A health clinic close by…close to a hospital. Just in case, you know…you never know. Being close to a hospital, that would be a big one.*” Mental health, particularly stress, was also raised in connection with QoL. Participants discussed the inherent challenges of being homeless and the lack of understanding that: “*People down here have been through hell. There is a lot that people are trying to run from. PTSD…being called a drunk, or a bum.*”

Numerous participants discussed how their addiction affected their health and QoL. Having access to alcohol, tobacco, and drugs was important to a number of both older and younger participants. While some expressed that drugs are important to their QoL and are a source of happiness (“*I wrote drugs on my list. It’s a terrible thing to say, but I’m an addict. You know, I get up every day looking for a fix*”), others wanted to reduce their use of these substances.

"*“Drugs make me happy, but I’m sad that I’m an addict. I know what it’s doing to me, the result is not good…yes, treatment options are important. For me, now, it is. Up to this point, drugs are really important to my quality of life…”*"

Several participants noted that drugs and alcohol provide the means to forget painful experiences in their past and the reality of the present. Some examples of painful experiences past and present include the loss of a spouse or close family member, the knowledge of contracting illnesses such as HIV, or managing chronic pain and other acute illnesses. Some individuals expressed that simply knowing where the next drink was coming from was their only source of security: ‘*Sometimes living in chaos is easier…better the devil you know…*’.

### Living conditions

Shelters were described as having both positive and negative effects on QoL. Most participants were grateful to be off the streets, to have a roof over their heads, and a warm place to sleep. Yet this view was often qualified with the caveat that “*if you have to stay in a shelter for a short time, you do, but it isn't a good thing in your life…it's kind of hard to live with a large group of alcoholics, drug addicts, and mental illness…*” Many participants commented on the restrictions shelters impose. Because of confidentiality issues (i.e., no visitors are allowed, one cannot call in and be told if an individual is living there), it is difficult to maintain contact with people who are outside of the shelter system. Many participants discussed the difficulty of not being able to see children or other family members. While shelters provide a safe place to sleep at night, many participants commented that they do not provide a source of respite: “*If you are tired in the afternoon you can't go and lie down… Even if you are allowed back in, all you can do is sit and stare at the walls.*” Other shelter-related issues discussed were: the need for more requirements around personal hygiene (i.e., require that clients shower because of bugs, infections, and body odor); concern for the acutely ill *(“… there are people at shelters who need help…don't let a man with serious mental illness live there forever…”),* conflicts with staff members, privacy issues, and being told when to wash and when to sleep. Other topics of discussion included the difficulty of finding bathrooms that are clean and safe, finding feces being left in the sink, addicts shooting up in group showers, needles being left behind by addicts, the threat and reality of being assaulted, and exposure to sexual acts (“*When the first thing you see after entering the refuge is someone jerking off in the shower in front of you, you really want to get out of this place…*”).

While some participants simply talked about getting off the street, many specified that they wanted to be able to move into a space of their own: “*An apartment is important, a place for me to go if things are not going well. If you’re in the street, you don’t have anywhere to go. You always end up in disgusting places.*” Some of the key attractions of clean, individual housing appeared to be the stability, privacy, and sense of pride that this would provide. Participants also expressed the need for spaces that are clean, safe, and have cooking facilities, a refrigerator, private bathrooms, and laundry facilities: “*Adequate housing in the sense that, I don’t think I’ve ever cooked for myself since I’ve moved into this housing. [It’s like] I’m living out of storage,*”; “*You need to eat in the morning to have enough energy to work a full day. How can you have food if you don’t have a fridge?*” and “*I got a stove in my room so…for me it’s a big deal…*”. Other participants expressed the value and sense of respect they derived from having their own laundry facilities.

"*“To be able to have clean clothes for my husband and me. To be able wash ‘em at least once a week. Cause it’s not nice when people smell and we’re very extremely clean people; we always want to have our clothes clean.”*"

Many participants expressed that single rooms are important in order to have one’s own space. Others found that the concept and conditions of single room occupancies (SROs) were distinct negatives for their QoL because of the isolation and lack of cleanliness.

"*“I don’t consider SROs homes. They are not. In Canada, I think it’s disgusting that we have all these single-room occupancies, all these people living in these slum hotels. ‘Cause you could actually have a cleaner environment in a nice tent.”*"

"*“[We can’t say]…oh let’s go over to your place and watch a movie, do you know what I mean? We don’t do that. We’re spread out all over the place.”*"

Several participants noted that they need access to certain resources because of specific conditions. For example, people who are HIV positive need access to clean drinking water and those with mobility issues (particularly older participants) would benefit from housing with an elevator.

Several participants also discussed the importance of the neighborhoods of the place where they were living or staying, particularly the feeling of being trapped and isolated within neighborhoods that offer few alternatives:

"*“And you get in your place and you are trying to change it, you’re getting slowly out of drugs. We used to do it [drugs] 24–7. That’s all we did. It’s too convenient in the downtown area. But if it’s still close to you, you’re going to do it here. But if you’re not going to do it, you got other things to get into, right?”*"

"*“Like in our building everyone is pretty well an addict because that’s it - we’re downtown. You can only change if you want to and if you want to, you gotta get a new place, you gotta get out of there. You gotta be away from that…”*"

Many expressed anger at witnessing extreme levels of violence, being exposed to emotional and physical abuse, and the level and degree of death and loss of members in their community.

"*“In some rooming houses, every room is a crack house. But sometimes you want to get away from that, go to work or go to a game. There are too many distractions, too much noise. Police are always there, people knocking on your door. People crashing at your place and never leaving, stealing your stuff.”*"

"*“I got sick of seeing people drop like flies, dead, getting rolled. Or you go visit someone and they’re laying there stiff. I can be talking to someone now. And like tomorrow – they’re gone. So much of that.”*"

Another aspect of living conditions that was frequently mentioned was food. Both the aesthetic and nutritional qualities of food were considered important to study participants. Nutrition was clearly a concern; participants mentioned the importance of eating enough, having balanced and nutritious meals, access to a variety of foods, and accommodations for dietary needs:

"*“Being able to get my meals for my husband and me. At least two meals a day because we never ate before. I want to get groceries in my house so that I have groceries there to cook for my husband and I because we are a team…”*"

There was a sense that satisfying food cravings, simple enjoyment of food, food choices, and perhaps even proper nutrition are luxuries that are beyond the reach of many study participants, yet many of them clearly wanted more than just enough food to survive. Participants talked about diets that lacked protein and vegetables, consisted heavily of fat and carbohydrates, and lacked variety.

### Financial situation

Some participants simply talked about having enough money to survive. For others, having no ownership of material goods meant they were stuck in their homeless status: “*Yes, that’s what I want, material stuffs. If you don’t have material stuffs in life, you are in the street.*” For some, money was linked to having choices, freedom, love, and recognition from society.

"*"…we are in the street and people already hate us because we don't have money. Try to get a girlfriend, or try to find a place in society [it’s impossible] if you don't have the material, if you don't have money, you have nothing."*"

"*“Money for me is freedom. When you have lots of money you have lots of responsibility. Most of the time people fail because of responsibility, But then again, if you don't have money it ends up the same, it is like a circle.”*"

For others, money was cited as a means to a specific goal, such as obtaining stable housing, being able to buy personal items (such as personal hygiene products), being able to buy things for one’s children, being able to go shopping when one wants to, and purchasing medications that are not covered by medical plans. Having money to better one’s self and to do things with and for one’s children was the topic of discussion for many participants.

Two further issues related to money that were commonly discussed were children being in jail or living in abusive circumstances. Participants felt helpless to offer any assistance as they saw money as being the only means to help their children (i.e., to post bail, escape traumatic foster homes, or resolve access and custody issues).

### Employment situation

A number of participants, particularly among the adults, indicated that they enjoyed working and felt better about themselves when they were employed. Both youth and adult participants stressed that they wanted jobs that were stable, legal, paid well, and involved something that they enjoyed doing. As with many Canadians, it was not just job availability that mattered, but job choices as well.

Many participants discussed the difficulty they faced finding work in the neighborhoods in which they lived. Many businesses are family owned and employers are reluctant to hire people who do not have established residences. The stigma of living in a shelter is difficult to overcome. Participants expressed that people believe that those who live in shelters do not want to work, are skeptical about the person’s ability to wake up in the morning and arrive on time, believe that such individuals are lazy and “*will drink [their] paycheck away*”, or must have done something wrong to be in their current situation. Many participants stated that hearing ‘no’ all of the time when looking for work is depressing and that “*there doesn’t seem to be any point to trying*.” Education was frequently mentioned in relation to participants’ employment situation, particularly as a requirement to find work: “*I think school is important, too, for a good quality of life. Nowadays it takes a grade 11/12 to work anywhere.”*

### Relationships

Virtually all of the participants discussed relationships, especially with family and friends, as being integral to their QoL. Both men and women talked about the importance of having contact with their children. For some participants, this contact was a source of joy whereas for others a lack of contact was a source of distress. Discussions about other family members, particularly parents, revealed more mixed responses. Many participants felt close to one or more family members and valued their relationships with them, but others said that family was not important. A few of the younger participants mentioned that they wished they could end conflicts with family and parents because the estrangement was draining and a source of stress. Some participants talked about their desire to have children someday whereas others expressed a complete lack of interest in having a family.

"*“It’s not worth having children in this society. If we have trouble looking after ourselves, we won’t be able to look after children. You have to learn how to take care of yourself first, you have to learn to like yourself, to trust yourself, to enjoy life, to be confident, to develop tools to be able to raise kids, to have a partner who want kids and who you love.”*"

‘Friends’ were described as people who are more than merely acquaintances.

"*“There are no friends in the street, only people you consume with, your real friends are those you had before you started consuming, they don't understand why you consume but if you stop they will still be your friends, whereas people you consume with are only there when you have dope.”*"

For some participants, friends appeared to take the place of family.

"*“…you can think about love but it will take you nowhere, I think of making new friends and that's all. If you think about love, your life is harder.”*"

Both the youth and adult participants stressed the importance of having caring friendships. One participant explained the significance of friendships by stating “*a lot of us are lonely*”.

### Recreational and leisure activities

A number of participants noted the importance of recreational and leisure activities for their QoL. These activities were described as providing a break from street life and being rewarding. Several participants mentioned the importance of being active, outdoors, and doing sports, whereas other participants talked about enjoying relaxing activities, such as reading, watching TV, or listening to music. Still other participants described their desire to travel and explore new places. An important aspect of recreational and leisure activities was the connection to others and prevention of loneliness: “*I like drawing, music, hanging out, and discussing with friends and playing hockey, I need that to be socially fulfilled*”. Other participants discussed the importance of being able to express themselves creatively through writing, making music, or painting.

### Broader themes

The six major content themes identified in this study were linked to several broader themes or concepts. Some of these broader themes include having choices, respect, some of the same rights as other members of society, and stability.

Being able to have choices or options for housing, work, and food was important to many participants. For most, it was not simply a matter of being grateful for any shelter or any job. For example, housing should meet particular needs and be clean and safe. It seemed that many participants were eager to have a home and not just a place to live. Likewise, a job should provide a sense of pride and more than a subsistence wage. Food should not just ensure survival, but it should vary and be nutritious and appealing. At the same time, others expressed ambivalence about whether these things would improve their QoL. For example, one participant noted that, given a choice, he would take an apartment and a good job but questioned whether this would improve his QoL. Another participant stated that living on the streets has been a blessing in disguise because he has no stress, no responsibilities, no worries, and no ambition.

Having a home and having an enjoyable, legal job were seen as two potential sources of self-respect. Having friends and positive interactions with family were also described as positive influences on one’s sense of self; these interactions make people feel worthy, loved, and valued. Many participants, particularly among youth, emphasized personal growth. In addition to self-respect, participants wanted respect and recognition from others. They told numerous stories about harassment and discrimination and were well aware of the negative stereotypes surrounding homeless individuals. Public perception of the homeless was a common topic. There were several participants who noted fundraising campaigns that showed homeless individuals eating out of garbage dumpsters. While they were aware of the need for funding social programs, they resented how homeless people were represented; "*We aren't that sort of person, but thanks for the money anyway!*"

The desire for choices and fulfilling home and work lives seemed to reflect a wish for some of the same rights and privileges enjoyed by other members of society – in essence, a desire to be seen as people and not just ‘homeless’. In many discussions, participants described themselves in relation to “*the citizens*,” wanting rights that “*the citizens*” have, and opportunities like “*the citizens*,” suggesting that they perceive themselves as being or being treated as something “*other*” than members of the society in which they live. Related to this sense of disenfranchisement from society, many participants discussed the need for, and the difficulties in obtaining, proper identification:

"*"To have all the cards you need for collecting welfare; you need a birth certificate, a health care card, a social insurance number, an address, and sometimes it takes a long time to [get] your social insurance number. In my case, I had to ask the local deputy because they did not want to give me a number."*"

A desire for autonomy and the ability to move around at will was expressed by many participants and may reflect earlier life experiences of being in government care or in institutional settings:

"*“Freedom, even though I pay rent, I need to be able to get out. If I feel like going somewhere, I'll hitchhike and leave. I don't want to feel stuck somewhere; it is my worst nightmare. I spent time in foster homes so now I need to feel I can get out of where I am.”*"

Interestingly, wanting a sense of stability was frequently mentioned by participants in conjunction with housing, “*to have a roof, to be in my own things, not having to find a place to sleep every single night*”. A number of participants commented on the lack of stability in their lives as a result of living in shelters. Several participants who had more stable housing noted that their housing situation provided a foundation from which to begin to address other issues in their lives. Many participants decried the lack of stable work, which would not only ensure a steady income but also provide structure to their lives. Indeed, stability and consistency in day-to-day living was clearly important, but lacking, for many participants and particularly for those living in shelters.

## Discussion

The homeless and hard-to-house participants in our study prioritized six major life area content themes that were important for their QoL: Health/health care, living conditions, financial situation, employment situation, relationships, and recreational and leisure activities. These themes were also related to several broader themes or concepts, which included having choices, respect, stability, and some of the same rights as other members of society.

The six identified major life area themes are quite consistent with QoL domains previously identified in various populations [15,21-23]. For example, the core QoL domains identified by experts and lay people in an international collaboration were physical health, psychological [state], level of independence, social relationships, environment, and spirituality/religion/personal beliefs [23]. Researchers used these domains for the development of the WHOQOL-100 (and its short form, the WHOQOL-BREF) and to assess QoL in the general population and individuals with a disease. As another example, Lehman [21] used relevant QoL literature and existing measures to identify nine life domains when developing a measure to assess QoL in chronically mentally ill individuals: living situations, family relations, social relations, leisure, work, finances, safety, health, and religion. Although the dimensions of these two conceptualizations are similar to the ones we identified with homeless and hard-to-house individuals, it is the specific details, life circumstances, or experiences within the domains that differ and are unique to homeless and hard-to-house individuals. Thus, while many QoL measures (such as the QOLI and WHOQOL-100) are used to assess QoL in homeless and hard-to-house individuals and will clearly touch upon relevant domains, it is the details within those domains that capture or do not capture this sub-population’s QoL experiences. The aspects that focus group participants identified as being important to their QoL (affordability of housing, access and cleanliness of bathing facilities, having a fridge or a stove, feelings of being stuck in one`s neighborhood, having balanced and nutritious meals and access to a variety of foods, living with or being surrounded by addiction and/or mental illness, concerns about infections caught from someone where one is living, and the stigma of being homeless) are particularly relevant or unique to this population. However, they are not reflected in the WHOQOL-100 or QOLI (or other measures that have been used to assess QoL or health-related QoL in this population, such as the SF-36 and SF-12). This illustrates the importance of developing instruments with input from the target population – in this case, homeless and hard-to-house individuals – to adequately, appropriately, and comprehensively assess their QoL.

Focus group participants also identified themes mentioned in other qualitative studies of homeless persons but in contexts other than QoL. Some of these studies focused on the adequacy of care [9] or homeless individuals’ experience of the health care system [14,24,25]. Participants noted similar themes such as lacking essential resources and its negative impact on health, encountering barriers to health care through being labeled and treated with disrespect, and feeling invisible to health care providers [24]. In the present study participants discussed mental health (particularly stress) in the context of the various challenges experienced when one is homeless, such as being viewed negatively by others. Additionally, some participants considered drug and alcohol use important because they felt it helped them forget, at least in the short-term, painful experiences that occurred in the past and sometimes in the present. Conversely, Grinman et al. [26] found that drug use contributed to poorer mental health status but not physical health status among homeless adults in Toronto, Canada.

Some focus group participants highlighted the importance of having their housing or shelter also meet specific health needs, such as providing easier access to health professionals and access to clean drinking water if living with HIV. This was similar to the findings of a mixed-methods study regarding the transitions out of homelessness associated with medical and substance abuse service use for a cohort of 174 chronically homeless street dwellers at high risk of death [27]. In this study, use of medical and substance abuse treatments did not have a favourable impact on housing attainment (with the exception of extended stays at homeless respite care) and the authors concluded that housing policies should reflect the need for integrating affordable housing and health services for chronically homeless street dwellers.

The focus group participants also identified various aspects of food as important to their QoL, particularly having balanced and nutritious meals as well as access to a variety of foods. Food insecurity has been found to be associated with lower access to ambulatory care and high rates of emergency department use and hospitalization [28]. Analysis of the data from the U.S. National Survey of Homeless Assistance Providers and Clients revealed that members of the homeless population did not uniformly experience hunger. Rather, complex patterns of food insecurity exist at the individual level based on the resources available and the barriers (e.g., substance abuse or mental health conditions) individuals face [29].

Although lack of employment is frequently cited as a key cause of homelessness, many homeless persons report having a job; the issue is that often these jobs do not provide adequate wages or benefits on which people can live [8]. Nonetheless, in a study of 471 homeless persons in California, the authors found that mental health, physical health and disability all played a significant role in the employment and program participation of the homeless and persons at risk for homelessness [8]. Employment was a concern for many study participants. For some, the stigma of living in a shelter was difficult to overcome and many thought it adversely affected their employment opportunities. Still, our participants reported feeling better about themselves when they were employed. Like many in society, they stressed the importance of job choices.

Participants identified relationships, social support and being able to have choices as being important to their QoL. They also wanted a sense of stability in both their housing and employment situations. Residing in shelters did not provide the opportunity for a daily routine with work or the housing stability they desired.

Participants also identified recreational and leisure activities as being relevant to their QoL. Participation in group activities provided a sense of belonging and connection, and being engaged in creative activities was described as a form of self-expression. Participants also emphasized the importance of being able to engage in relaxing activities as a balance to the stressors of providing for themselves. They associated living on the street with considerable time demands: time spent obtaining food (e.g., standing in line at a food bank), securing housing for the night (e.g., lining up to get a bed in a shelter), finding transportation (e.g., when public transport fare is not affordable), and making money (e.g., collecting bottles to exchange for refund).

Strengths of this study include the relatively large sample size for focus group research with multi-site sampling from four cities. Through the focus groups, we were also able to probe more deeply into the participants’ subjective perspectives on the QoL themes and issues raised. This study also had some limitations. Focus group moderators differed across cities and therefore the training and implementation of the focus groups may have differed somewhat. The sample lacked some cultural diversity and persons from immigrant communities who were experiencing housing instability or homelessness may not have volunteered to participate. There are also limitations with respect to fully understanding the representativeness of our sample due to missing data (demographics).

## Conclusions

In summary, homeless and hard-to-house adults and street youth from multiple sites in Canada identified six key content themes - health/health care, living conditions, financial situation, employment situation, relationships, and recreational and leisure activities - as being important to their QoL. These themes are similar to the life areas raised in other research but differ from the general population and other subgroups in terms of the details, examples, circumstances, and experiences of those themes in a way that reflects the unique contexts and life experiences of individuals who are homeless or hard-to-house. For example, while typically housed individuals may care about aesthetics, layout, size, functionality, and market value of their housing, homeless and hard-to-house individuals may find such issues irrelevant when they are (i) living in a park, tent, or shelter, or (ii) dealing with bed bugs, no appliances, shared bathrooms, or mentally ill or severely addicted neighbours. The six identified themes were also linked to the broader and less tangible themes of having choices, respect, stability, and the same rights as other members of society.

Our findings are relevant to generating measures that capture QoL specific to this population. Given the increased mortality and morbidity associated with homelessness [30-33] and that effective interventions are often complex with components of both social policy and health care, an instrument that can capture the intended effects on QoL will be crucial for rigorous scientific evaluation of such interventions. Currently available QoL instruments may be limited in scope and not include specific aspects of life areas that are important to individuals who are homeless limiting their ability to detect change. Based on our results, we are developing the Quality of Life for Homeless and Hard-to-House Individuals (QoLHHI) instrument to assess QoL among homeless and hard-to-house persons [34]. Furthermore, the development of programs and policies that are informed by and address these life areas may be more effective in improving the life situation for persons who are homeless and hard-to-house.

## Competing interests

The authors declare that they have no competing interests.

## Authors’ contributions

AP and AMH were involved in study conception and design, analysis and interpretation of the data, and drafting of the manuscript. LBR co-moderated the focus groups in Vancouver and was involved in the analysis and interpretation of the data and drafting of the manuscript. AMG and MC were involved in the interpretation of the data and drafting of the manuscript. All authors read and approved the final manuscript.
